# Hook, Line, and Thinker: Seafood Nets Benefits for Neurocognitive Development

**DOI:** 10.1016/j.advnut.2025.100415

**Published:** 2025-04-25

**Authors:** J Thomas Brenna

**Affiliations:** 1 Department of Pediatrics, Dell Pediatric Research Institute, University of Texas at Austin, Austin, TX, United States; 2 Department of Chemistry, Dell Pediatric Research Institute, University of Texas at Austin, Austin, TX, United States; 3 Department of Nutrition, Dell Pediatric Research Institute, University of Texas at Austin, Austin, TX, United States

The old saying “seafood is brain food” is among the most persistent nutritional memes. It originated from a casual observation made nearly 140 y ago by the renowned naturalist Louis Agassiz, who noted that both fish and brains are rich in phosphorus. When Mark Twain learned of this comment, he humorously remarked to a group of aspiring authors that, based on their writing samples, they would need to consume “2 middling-sized whales” to achieve professional levels. As the most beloved humorist of his era, Twain’s quip rapidly disseminated across the globe, embedding itself in public consciousness [1]. New Yorkers even flocked to fish markets, hoping that increased seafood consumption might unlock their latent brilliance [2].

Whether or not phosphorus plays a direct role in brain development, the enduring appeal of seafood as brain food feeds an appetite to optimize neurocognitive function through diet. In recent issues of *Advances in Nutrition: An International Journal*, researchers at Texas A&M University have presented 5 comprehensive systematic and scoping reviews that critically examine both the beneficial and potentially adverse effects of seafood consumption on neurocognitive development. The publication is particularly timely given that the FDA’s final rule—effective February 2025—allows seafood, among other foods, to be labeled healthy [3]. Spoiler alert: the news here continues to be good.

Two systematic reviews by O’Connor et al [4,5] examined the effects of seafood consumption on neurocognitive development—one focusing on the prenatal period (pregnancy/lactation) and the other on childhood. The findings further confirm that seafood offers real benefits, even though regulatory and policy frameworks have yet to fully integrate these scientific insights.

For maternal seafood intake during pregnancy, the evidence indicates that higher consumption is associated with improved behavioral outcomes in offspring from birth through age 19 (spoiler alert: those old mercury fears are overblown) [4]. The review, which assessed 40 studies—primarily prospective cohort studies—spanned a variety of neurodevelopmental domains, with a particular focus on behavioral metrics. For instance, maternal seafood intake of 4–16 oz/wk was consistently linked to better child behavioral outcomes. Notably, the systematic review used rigorous quality assessments, including sensitivity analyses that reinforced the robustness of these associations even when studies at high risk of bias were excluded. This comprehensive analysis underscores the potent neurodevelopmental benefits of seafood, likely mediated by the high concentrations of ω-3 fatty acids and other essential nutrients present in fish.

The childhood consumption review, analyzing 18 studies, found that children who consume more seafood—primarily fatty fish—demonstrate enhanced cognitive development between the ages of 0 and 18 y [5]. Beneficial effects were observed with fish intakes of 5–11 oz/wk, aligning closely with current dietary recommendations. The studies, many of which were short-term randomized controlled trials (RCTs) conducted in Northern Europe comparing fatty fish with meat/poultry interventions, consistently reported modest yet reproducible improvements in cognitive scores. Furthermore, this review incorporated both RCTs and prospective cohort studies, allowing for a nuanced evaluation of seafood’s role in cognitive enhancement; the observed benefits are likely attributable to the ω-3 fatty acids that support neuronal membrane structure and synaptic function. These findings suggest that even moderate increases in seafood consumption during childhood can yield measurable cognitive benefits.

But what of contaminants? Three systematic reviews examined seafood’s main contaminant concerns—mercury, polychlorinated biphenyl (PCBs), and lead, and the data are more reassuring than alarming. For PCBs, a systematic review of 7 studies by Balalian et al [6] found only minimal associations between PCB exposure from seafood during pregnancy/lactation and child growth outcomes. Although a tiny negative correlation with birth weight (*r* = −0.07) was observed, no significant associations emerged for birth length or head circumference; the authors noted that current PCB exposure levels are substantially lower than those observed during historical contamination events, indicating that modern seafood consumption poses little PCB risks.

The systematic review by Balalian et al. [7] on lead examined 4 studies but found no clear evidence that lead exposure from seafood during pregnancy adversely impacts neurodevelopment. Although 1 study identified a weak negative association with motor development at 12 mo, this effect was no longer evident by 36 mo [7]. Moreover, the review emphasized that seafood contributes only ∼3% of typical lead exposure, further mitigating concerns about its impact on child development.

Finally, the toxicant scoping review by Trivedi et al [8] identified 81 studies examining various contaminants, with mercury being the most frequently studied (*n* = 49 maternal exposure studies). However, few toxicant–outcome pairs had sufficient evidence to draw firm conclusions; the authors identified only 12 maternal and 1 child toxicant–outcome pairs with adequate data for systematic review.

Taken together, these reviews paint a compelling picture: the cognitive benefits of moderate seafood consumption—both during pregnancy and childhood—appear to outweigh the theoretical risks posed by contaminant exposures. Moreover, the negative associations observed were generally small and inconsistent.

Numerous large-scale reviews on seafood nutrition and its health benefits have appeared recently, notably by FAO–WHO [9,10] and NASEM [11]. Possibly, the largest is a 1000+ page 2022 Norwegian study that concluded “…that the benefits from increasing fish intake to the recommended 2 to 3 dinner courses per week (corresponding to 300–450 grams [10–16 oz], including ≥200 grams [7 oz] fatty fish in adults) outweigh risks for all age groups” [12]. The FAO–WHO similarly state that “maternal fish consumption during pregnancy is associated with improved offspring neurological development….” Years of research in this area [13,14] have led the author to concur in these opinions. A key factor for these evaluations is to consider the study of seafood consumption itself, rather than its individual component nutrients and contaminants, as the unit of measure.

These consistent findings have important implications for public health messaging. Historical emphasis on contaminant risks has discouraged beneficial seafood consumption, contributing to current United States intake levels that remain well below recommendations: <6% of Americans eat seafood twice weekly. Ambiguous government messaging deters seafood consumption during pregnancy [15]. A nuanced, consumer-tailored approach to dietary guidance is likely to be most effective [16]. The evidence strongly suggests that public health initiatives should promote increased seafood consumption, particularly during pregnancy, as insufficient intake is more detrimental to neurodevelopment than the modest contaminant risks. Maintaining reasonable precautions regarding specific high-contaminant options remains prudent as it is for all foods, but such advice should not overshadow the established benefits of seafood.

The bottom line? It is time to move past an excessive fear of seafood contaminants and focus on integrating more beneficial fish into both maternal and child diets. The cognitive benefits are real, while the contaminant risks are overstated—at least within the context of typical consumption patterns in developed nations.

## Author contributions

The sole author was responsible for all aspects of this manuscript.

## Funding

The author reported no funding received for this study.

## Conflicts of interest

The author reports no conflicts of interest.
